# Use of non-medical cannabis in epilepsy: A scoping review

**DOI:** 10.3389/fneur.2023.1132106

**Published:** 2023-03-06

**Authors:** Jimmy Li, Cassandra C. Areal, Dènahin Hinnoutondji Toffa, Daphné Citherlet, Charles Deacon, Didier Jutras-Aswad, Mark Robert Keezer, Dang Khoa Nguyen

**Affiliations:** ^1^Neurology Division, Centre Hospitalier de l'Université de Sherbrooke (CHUS), Sherbrooke, QC, Canada; ^2^Neurosciences Axis, Centre de Recherche du Centre Hospitalier de l'Université de Montréal (CRCHUM), Montreal, QC, Canada; ^3^Department of Neurosciences, Université de Montréal, Montreal, QC, Canada; ^4^Department of Psychiatry and Addictology, Université de Montréal, Montreal, QC, Canada; ^5^School of Public Health, Université de Montréal, Montreal, QC, Canada; ^6^Neurology Division, Centre Hospitalier de l'Université de Montréal (CHUM), Montreal, QC, Canada

**Keywords:** cannabis (marijuana), seizure, epilepsy, tetrahydrocannabinol (THC), cannabidiol (CBD), scoping review

## Abstract

**Objective:**

The use of medical cannabis among people with epilepsy (PWE) has been garnering increasing interest. In this scoping review, we aimed to summarize the literature on recreational/non-medical cannabis (NMC) use in PWE, focusing on the experience, habits, and beliefs of PWE regarding NMC.

**Methods:**

Four databases (OVID Medline, OVID Embase, Ovid APA PsycInfo, and Web of Science) were searched for studies describing NMC use in PWE. NMC was defined as cannabis products procured from sources other than by prescription. Studies that consisted in original research and that detailed the experience, habits, and/or beliefs of PWE regarding NMC use were included in the analysis. Data pertaining to study identification, demographics, NMC use, and epilepsy characteristics were extracted. Descriptive statistical analyses and reflexive thematic analyses were performed to map these data.

**Results:**

In total, 3,228 records were screened, and 66 were included for analysis: 45 had mainly adult samples, whereas 21 had mainly pediatric samples. Most studies were published after 2010, originated from the USA, and were cross-sectional. The median number of PWE using cannabis in these studies was 24.5 (1–37,945). No studies showcased elderly PWE, and most had predominantly Caucasian samples. The lifetime prevalence of NMC use in PWE was variable, ranging between 0.69 and 76.8%. Factors frequently associated with NMC use in PWE were male sex, younger adult age, and lower education status. Children with epilepsy took NMC primarily for seizure control, using high CBD/THC ratios, and only orally. Adults with epilepsy took NMC for various reasons including recreationally, using variable CBD/THC ratios, and predominantly through smoking. The majority of PWE across all studies perceived that NMC aided in seizure control. Other aspects pertaining to NMC use in PWE were rarely reported and often conflicting.

**Conclusion:**

The literature on NMC use in PWE is sparse and heterogeneous, with many salient knowledge gaps. Further research is necessary to better understanding the experience, habits, and beliefs of PWE pertaining to NMC.

## 1. Introduction

Cannabis, also commonly referred to as marijuana, is most often derived from the *Cannabis sativa* plant and has been used for millennia for recreational, medicinal, and spiritual purposes (1). Historically, cannabis was categorized as an illegal substance in many Western countries (2). In the last decades, however, cannabis has seen a rise in interest in developed countries due to its potential medicinal qualities (2, 3). Research on the medicinal properties of cannabis has pushed many administrative bodies to authorize prescribing medical marijuana for certain disorders, such as epilepsy (3). In the last years, an increasing number of jurisdictions have either decriminalized or legalized non-medical cannabis (NMC) use, making cannabis more available for the general population (2).

Although there is debate on how many active compounds are present in cannabis, *trans*-Δ-9-tetrahydrocannabinol (THC) and cannabidiol (CBD) are the most described. The psychoactive properties of cannabis are mostly mediated by THC (1). While CBD has clearly been shown to have anti-seizure properties, contradictory pro-convulsant and anti-seizure effects have been reported for THC (4, 5). Four landmark randomized controlled trials (RCTs) have notably demonstrated the anti-seizure benefits of CBD in Lennox-Gastaut syndrome, Dravet syndrome, and tuberous sclerosis. In response, the US Food and Drug Administration (FDA) approved Epidiolex, an oral CBD solution, for the treatment of these three syndromes (3, 6–9). Although Epidiolex is not yet approved in many parts of the world, the evidence surrounding the anti-seizure properties of CBD has prompted an increased demand for high CBD and low THC cannabis oils among individuals with refractory epilepsy (4).

Although medical cannabis is now more readily available in many developed countries, its price point limits its use by many people with epilepsy (PWE). In the US, for instance, Epidiolex is estimated to cost tens of thousands of dollars yearly (4). As recent research on cannabis in epilepsy has mostly focused on medical cannabis despite it not being readily available for PWE in many countries, the following question arises: what is known about the use of NMC in PWE? The prevalence of PWE consuming NMC is not well-described, although some studies report that more than half of PWE consume cannabis, with many basing their consumption on cannabis' role in seizure control (10, 11). This reasoning is not trivial since NMC (with often non-negligible THC content) has not been shown to be an efficacious anti-seizure agent and, while it is used without significant negative consequences by many, it has been associated with various harmful health effects (12). Achieving a better understanding of the various factors underpinning NMC consumption in PWE should be a research priority, especially given its potentially hazardous effects on health and the currently limited availability of medical cannabis in many countries.

## 2. Methods

The protocol for this review was registered on the Open Science Framework platform (10.17605/OSF.IO/C5E74) and follows the Preferred Reporting Items for Systematic Reviews and Meta-Analyses extension for Scoping Reviews (PRISMA-ScR) reporting guideline (13). A scoping review is defined as “a form of knowledge synthesis that addresses an exploratory research question aimed at mapping key concepts, types of evidence, and gaps in research related to a defined area or field by systematically searching, selecting, and synthesizing existing knowledge” (14). The aim of broadly mapping a body of literature is one of the key aspects differentiating a scoping review from a systematic review, which rather aims to answer a set of defined questions by critically appraising the literature. Though both types of review require a similar rigorous, transparent methodology, a scoping review will usually screen for multiple different study designs and will not task itself with quality assessment (15).

### 2.1. Objectives

This scoping review sought to answer the following research question: “What is known about the consumption of NMC in PWE?” More broadly, we aimed to summarize the nature, extent, and range of the available research on the subject, identify relevant knowledge gaps, and offer recommendations for future research (16). NMC was defined as all cannabis products, in any form (e.g., whole plant or oil), that are procured from sources other than by prescription from a medical professional (e.g., legal market, if applicable, or illicit vendors). As such, even if a cannabis product was used by an individual for a “self-medicating” purpose (e.g., for seizure control), if this product was not prescribed by a medical professional, it was still considered NMC. The term “recreational cannabis,” which can be thought of as interchangeable with NMC, was not employed in this review, as it may infer that the cannabis product is used only recreationally, whereas it may in fact be used mainly for its perceived health benefits.

### 2.2. Search strategy

All authors revised and contributed to the search strategy. A health sciences librarian reviewed the search strategy and offered adjustments, and a consensus was eventually reached to search the following databases: OVID Medline, OVID Embase, OVID APA PsycInfo, and Web of Science (17). Free-text terms were combined in various manners with controlled vocabulary terms (when applicable) to search for research reporting on humans. These search terms were inspired from previous research investigating strategies for identifying citations on epilepsy and cannabis (18, 19). The full search strategy, including sources of gray literature, is available in Supplementary Table S1. No restrictions were placed on language or time of publication. Online translation services were employed when feasible for studies written in languages other than English, French, and Spanish. We employed a “snowballing” method by which the reference lists of up to 20 selected literature reviews on cannabis use in epilepsy were manually reviewed for additional works of interest. All citations were imported in the Covidence online platform, which was used for the study selection phase. Duplicate citations were automatically removed by the Covidence platform, and duplicates that remained were manually identified during the study selection phase.

### 2.3. Study selection

Two reviewers (J.L. and C.C.A.) independently screened titles and abstracts on the Covidence platform for works pertaining to the use of NMC in PWE. At this stage, citations were included if they explicitly described NMC consumption in PWE or if they described cannabis consumption in PWE without enough information to determine if the cannabis was medical or non-medical. Citations explicitly only describing medical cannabis use in PWE (e.g., studies in which patients with epilepsy are offered CBD products by the investigators), only describing the drug mechanisms of cannabis, only involving non-human subjects, or consisting in duplicates were excluded. No restriction was placed on study type. Full-text articles were then independently reviewed by the same two reviewers (J.L. and C.C.A.) using the Covidence platform. At this stage, citations were included if they described the experience, habits, and/or beliefs of PWE in relation to NMC use. Citations were excluded if they only pertained to medical cannabis use, if they only described the drug mechanisms of cannabis in epilepsy, if they consisted in duplicate citations, or if they were not original research. Any disagreement between the two reviewers were resolved through discussion, with third party (D.H.T.) intervention as necessary.

### 2.4. Data extraction

Data were independently extracted from the works having passed the selection phase by one reviewer (J.L.) using an Excel spreadsheet. A second reviewer (C.C.A.) verified the data extraction form, and disagreements were handled through discussion with a third party (D.H.T.) when needed. The following information was extracted when available:

a. Publication identification: first author, title, publication year, publication origin, journal/conference of publication, publication type, and potentially significant funding/conflicts of interest.
b. Study characteristics: sample, study aim, sample type, number of study participants in total (e.g., number of PWE in total if only a subset used NMC), and number of PWE using NMC.
c. Demographics (of PWE using NMC): age, sex, education status, comorbidities, socio-economic standing, and marital status.
d. Epilepsy data: age of onset, epilepsy type (focal-onset vs. generalized-onset), epilepsy syndrome, and antiseizure medication (ASM) use.
e. NMC data: experience with NMC (prevalence of consumers among PWE or of epilepsy among consumers, factors independently associated with consumption, dependency on NMC, consequences of consumption on physical, mental, and social spheres), habits with NMC (type of NMC consumed, administration method, dose of CBD and THC in NMC, frequency of consumption, time since start of consumption, age at first consumption, source of acquisition, product pricing), and beliefs in relation to NMC (goals for consumption, information sources, general knowledge of cannabis, opinion on regulatory policies, and perceived benefits and drawbacks of consumption on seizure control or other aspects of livelihood).

### 2.5. Data synthesis

Data were synthesized using Excel. Our analysis comprised a numerical component, wherein data were synthesized using descriptive statistical methods, and a thematic component, wherein data were qualitatively analyzed for themes using a reflexive approach (20). Descriptive statistical analyses were used to synthesize findings from adult studies and pediatric studies separately. Numerical data are presented as medians (range) and count (proportions), where appropriate. All descriptive statistical analyses were performed using R.

## 3. Results

**Figure 1:**
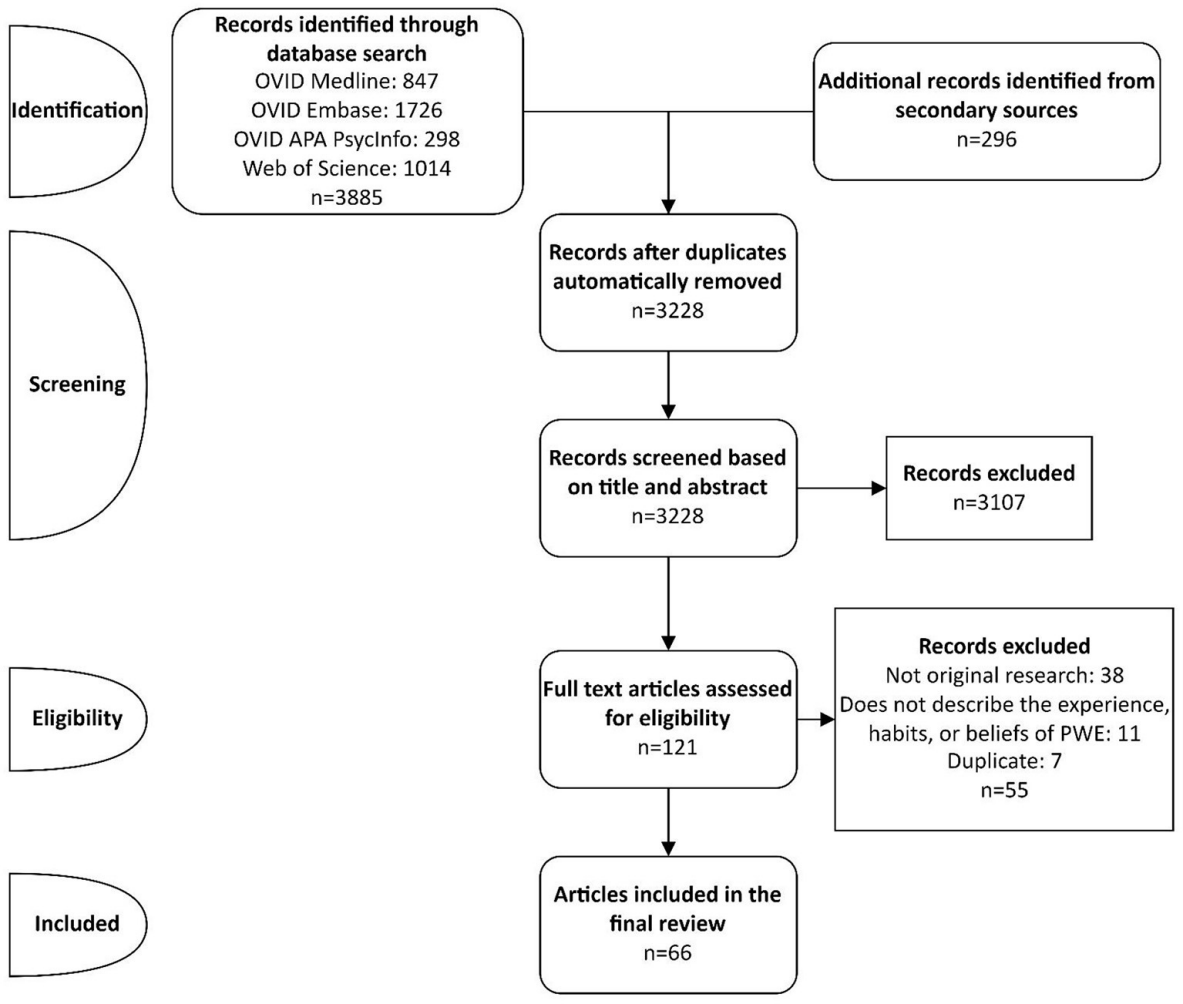
Data selection flowchart.

A total of 4,182 records were identified using the search strategy in Supplementary Table S1. With 953 records being flagged as duplicates by Covidence, 3,228 records underwent the title/abstract screening phase. A total of 121 records passed this phase and underwent full-text screening. Of these records, the reference lists of 20 selected literature reviews were manually examined for additional works of interest. No additional works of interest were detected. With 55 records being excluded from the full-text screening phase, 66 records were included in the final analysis (2, 4, 10, 11, 21–82). The data selection flowchart is presented in Figure 1.

**Figure 2:**
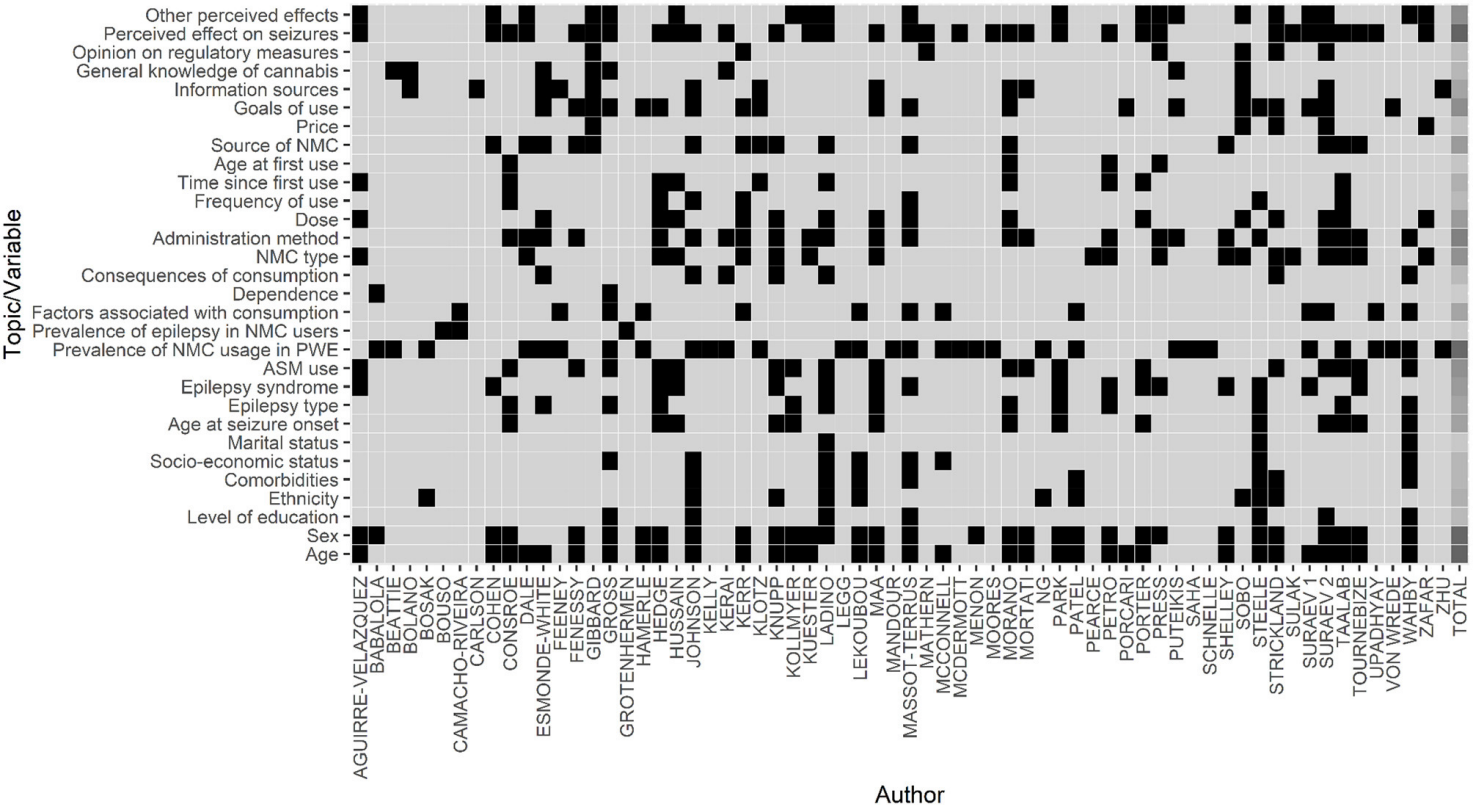
Heatmap of topics discussed in each included study. This figure consists of a heatmap summarizing which topics were discussed in each included study. The x-axis represents the studies that were included in this scoping review, as identified with the first author. The y-axis represents all the topics that we searched for in each study (see Section 2.4. Data extraction for more information). A black square at the junction between a study (x-axis) and a topic (y-axis) indicates that that topic was discussed in that study. A light gray square at that same junction would indicate that that topic was not discussed in that study. The last column represents the sum of the times each topic was discussed by all authors. In this last column, a square takes on a darker shade of gray as the topic is more “popular” (i.e., it was discussed in more publications). For instance, the perceived effect of seizures was a topic that was discussed in many studies, whereas the age at first use of cannabis was rarely discussed. ASM, anti-seizure medication; NMC, non-medical cannabis; PWE, people with epilepsy.

Table 1 lists the studies included in this review and provides a summary of their sample and primary aim. Table 2 presents in detail the publication identification and study characteristics of all 66 included studies whilst providing separate information for adult and pediatric studies. Supplementary Table S2 presents how many times each variable of interest (demographic, epilepsy, and NMC data) is explored across all included studies and between adult and pediatric studies. Figure 2 consists in a visual summary of the topics/variables touched upon by each study, taking the form of a “heatmap.” An in-depth exploration of all variables of interest measured across all included studies is provided in Supplementary Table S3.

**Table 1:** Summary of the study types, samples, and aims of included studies.

**References**	**Year**	**Study type**	**Sample**	**Aims**
Aguirre-Velazquez (21)	2016	Cross-sectional	Children with refractory epilepsy taking NMC	To survey medicinal use of CBD in children with refractory epilepsy in Mexico
Babalola (22)	2013	Cross-sectional	Patients with epilepsy at Nigerian clinic	To survey substance use in PWE in a Nigerian hospital and evaluation socio-demographic variables associated with substance use
Beattie et al. (23)	2017	Cross-sectional	Patients admitted to pediatric EMU in Alabama	To investigate the use of complementary alternative medicine and the influence of religion in children admitted to EMU
Bolano et al. (24)	2018	Cross-sectional	Patients with epilepsy, Parkinson's, or MS	To investigate the use of cannabis in patients with neurological disorders
Bosak and Slowik (25)	2019	Cross-sectional	Adult PWE seen at epilepsy clinic (no PNES)	Determine the prevalence, reasons, and factors influencing the use of complementary alternative medicine in PWE
Bouso et al. (26)	2019	Cross-sectional	Therapeutic members of social cannabis clubs with chronic illnesses	To assess quality of life, mental health, and personality variables, as well as the description of patterns of cannabis use, in people self-medicating with cannabis
Camacho-Riveira et al. (27)	2021	Cross-sectional	Cannabis users with or without cancer	To identify behaviors with cannabis use among cancer survivors vs people without cancer
Carlson (28)	2014	Cross-sectional	Medical professionals	To survey medical professionals who treat PWE on their opinion on cannabis and the use of cannabis in their patients
Cohen et al. (29)	2020	Cross-sectional	PWE taking artisanal and/or pharmaceutical CBD who had CBD serum levels	To investigate if CBD levels are higher in patients taking pharmaceutical vs artisanal CBD
Consroe et al. (30)	1975	Case report	PWE 1 case	To describe the possible beneficial effects of cannabis in epilepsy
Dale et al. (31)	2021	Cross-sectional	Participants in the International CDKL5 Disorder Database	To describe caregiver perceptions of efficacy and safety of cannabis in individuals with CDKL5 disorder
Esmonde-White et al. (32)	2021	Cross-sectional	PWE	To investigate if cannabis is used, use habits, and perceptions of cannabis, in PWE
Feeney (33)	1976	Cross-sectional	PWE followed at American medical center	To examine the extent of cannabis use in PWE
Fennessy et al. (34)	2018	Case report	PWE 1 case	To discuss the issues surrounding cannabis prescription in Ireland
Gibbard et al. (35)	2021	Qualitative	Parents of < 18 y/o patients who currently or previously used any form of cannabis for medical purposes	To explore family experiences using medical cannabis for children with severe conditions in Canada
Gross et al. (36)	2004	Cross-sectional	Adult PWE from Canadian epilepsy clinic	To determine the prevalence of and reasons for cannabis use in PWE
Grotenhermen and Schnelle (37)	2003	Cross-sectional	Members of the German Association for Cannabis as Medicine (ACM)	To survey ACM members on their experiences with the medical use of cannabis products, comparisons between natural cannabis and THC, the attitude of their doctors and health insurances toward THC.
Hamerle et al. (38)	2013	Cross-sectional	Adult PWE seen at German outpatient clinic	To investigate the prevalence of cannabis consumption in PWE, factors associated with consumption, and effects on epilepsy
Hegde et al. (39)	2012	Case series	PWE 2 cases	To offer cases of seizures documented by vEEG in the setting of cannabis discontinuation
Hussain et al. (40)	2015	Cross-sectional	Members of the Infantile Spasms Community and the Lennox-Gastaut Foundation who administered cannabis products to their children	To document the experiences of children with infantile spasms and/or Lennox-Gastaut syndrome who have been treated with CBD-enriched cannabis preparations
Johnson (41)	2021	Cross-sectional	Adults with epilepsy currently living in the community	To examine cannabis use among a sample of adults with epilepsy and assess its association with domains of QOL
Kelly and Chung (42)	2012	Cross-sectional	Patients seen at an epilepsy clinic	To investigate the use of supplements and complementary medicine in PWE
Kerai et al. (43)	2018	Cross-sectional	Adults with epilepsy or caring for someone living with epilepsy in Western Australia (online group)	To investigate the perceived needs for medical cannabis in the management of epilepsy of adults with epilepsy and caretakers of a person with epilepsy in Western Australia
Kerr et al. (10)	2019	Cross-sectional	Adults > 21 y/o with epilepsy seen at epilepsy clinic	To ascertain how patients with epilepsy at a tertiary care clinic in Oregon are using cannabis outside of the medical system
Klotz et al. (44)	2020	Cross-sectional	Caregivers of PWE from 5 epilepsy clinics or online organizations	To gain information about parental attitude to CBD, as well as expectations and knowledge of CBD for treatment of their child's epilepsy
Knupp et al. (45)	2019	Cohort	Patients < 21 y/o with refractory epilepsy planning to start CBD product	To follow children prospectively when families chose to use oral cannabis extracts for treatment of refractory epilepsy to better characterize response rate, AE and product types in use
Kollmyer et al. (46)	2019	Case series	2 PWE	To report two deaths of patients whose reliance on self-determined therapy with cannabis for seizure prevention was not benevolent
Kuester et al. (47)	2016	Case series	11 PWE taking oral cannabis extracts followed at Chilean hospital	To report preliminary findings in a series of patients with different types of refractory epilepsy treated with oral cannabis extracts
Ladino et al. (48)	2014	Case series	18 PWE holding a prescription of medicinal marijuana in a Canadian epilepsy center	To investigate sociodemographic and clinical characteristics of a group of PWEs with a formal prescription for medicinal marijuana
Legg et al. (49)	2012	Cross-sectional	Adults assessed at a Canadian First Seizure Clinic	To investigate self-reported cannabis use in all patients referred for suspected first seizure
Lekoubou et al. (50)	2020	Cross-sectional	Adults with epilepsy in the National Inpatient Sample database (2006-2014)	To ascertain recent nationwide prevalence, trends, and psychiatric diagnoses associated with CUD among epilepsy patients
Maa and Figi (51)	2014	Case report	1 PWE	To describe the case of Charlotte Figi
Mandour and Hazim (83)	2021	Cross-sectional	Patients with refractory epilepsy compliant to ASM seen at Egyptian epilepsy clinic	To study the etiology of non-response to ASM by estimating their serum levels and screening of drugs and substance abuse in patients with resistant epilepsy
Massot-Tarrus and McLachlan (11)	2016	Cross-sectional	Adult patients admitted to Canadian EMU	To determine the prevalence of cannabis use and assess the perceived effects in intractable epilepsy patients compared to those not found to have epilepsy
Mathern et al. (52)	2015	Cross-sectional	Health professionals and PWE to whom was advertised the survey	To survey the opinions on the use of cannabis for PWE
Mcconnell et al. (53)	2013	Cross-sectional	PWE seen at American epilepsy clinic	To examine the general acceptance and use patterns of complementary and alternative medicine in an epilepsy clinic
Mcdermott et al. (54)	2021	Cross-sectional	PWE seen by an epileptologist at American hospital	To assess the frequency of use and mode of use of cannabinoid compounds and patient perception of efficacy of cannabinoid compounds for seizure management and effect on QOL
Menon et al. (55)	2016	Cross-sectional	Women with epilepsy seen in private and public setting in Jamaica	To assess the practices according to accepted standards of care
Moores et al. (56)	2018	Cross-sectional	Adult PWE seen at Canadian hospital	To survey cannabis use in PWE
Morano et al. (57)	2016	Case report	1 PWE	To report the case of a patient with focal epilepsy who used cannabis as self-medication after the failure of countless pharmacological/surgical treatments
Mortati et al. (58)	2007	Case report	1 PWE	To present a patient whose medically intractable symptomatic focal epilepsy markedly improved with cannabis and review the literature regarding cannabis and epilepsy
Ng et al. (59)	1990	Case-control	People over 15 y/o admitted for first seizures at American hospital	To report the relative risks of heroin, marijuana, and cocaine use for new-onset seizures
Park and Roth (60)	2015	Case report	1 PWE	To report a patient with intractable epilepsy whose spike count on continuous EEG monitoring correlated with inhaled cannabis use
Patel et al. (61)	2019	Cross-sectional	Adults with epilepsy in the National Inpatient Sample database (2010–2014)	To compare the incidence of epilepsy between patients with CUD and without CUD, to examine the characteristics of hospitalized PWE with CUD, and evaluate the association between CUD and epilepsy hospitalization
Pearce et al. (62)	2014	Cross-sectional	Self-identified medical cannabis users on online platforms	To compare C. indica and C. sativa in terms of health symptoms, conditions, purpose, route, and trust in product
Petro (63)	2015	Case series	2 PWE	To present two cases demonstrating the safety and efficacy of cannabis use to treat epilepsy and anorexia
Porcari et al. (64)	2018	Cross-sectional	PWE with epilepsy using artisanal CBD-containing products in the Vanderbilt Synthetic Derivative database	To define the efficacy of artisanal CBD preparations in PWE with epilepsy
Porter and Jacobson (84)	2013	Cross-sectional	Parents of children with epilepsy who support use of cannabis to treat children (Facebook group)	To explore the use of cannabidiol-enriched cannabis in children with treatment-resistant epilepsy
Press et al. (65)	2015	Cohort	Children with epilepsy who have trialed oral cannabis extracts seem at American hospital	To report on the experience of a cohort of pediatric patients with epilepsy who were given oral cannabis extracts
Puteikis and Mameniskiene (66)	2020	Cross-sectional	Adult PWE visiting Lithuanian hospital	To evaluate whether the topic of using cannabis in epilepsy is relevant among adult PWE and assess the attitudes for having a history of consumption or being inclined to start consumption
Saha et al. (67)	2006	Cross-sectional	PWE admitted to African hospital with post-ictal complications	To evaluate substance use in PWE
Schnelle et al. (68)	1999	Cross-sectional	People from Germany, Austria, and Switzerland surveyed by the Association for Cannabis as Medicine	To survey cannabis use in PWE
Shelley et al. (69)	2016	Case report	1 PWE	To present a case of a parent who purchased CBD oil on the internet and administered it to her child
Sobo (70)	2017	Qualitative	American parents using, interesting in using, or who have used cannabis for a child's seizures	To explore how parents of children with epilepsy learn about, procure, dispense, and monitor cannabis use for their children
Steele (71)	2020	Cross-sectional	Adult PWE using cannabis through online recruitment source	To examine the potential association between mood disturbance and QOL as moderated by cannabis use in PWE
Strickland et al. (72)	2021	Cohort	PWE recruited using patient registries from Realm of Caring Foundation and social media posts	To evaluate associations of artisanal CBD product use with QOL, mental health, healthcare utilization, and epilepsy-specific outcomes within a large, observational cohort of PWE
Sulak et al. (73)	2016	Cross-sectional	PWE seen at American children's hospital and private cannabis medicine practice	To report the retrospective data on efficacy and AE of artisanal cannabis in patients with refractory epilepsy
Suraev et al. (75)	2017	Cross-sectional	PWE (survey promoted online by Epilepsy Action Australia)	To survey adults with epilepsy and parents/guardians of a PWE on cannabis use
Suraev et al. (74)	2018	Qualitative	Families who had a child aged < 16 y/o with epilepsy	To explore Australian families' experience with and perspectives on cannabis extract use for childhood epilepsy
Taalab et al. (76)	2019	Cross-sectional	PWE seen at Egyptian clinic with no substance abuse other than cannabis	To investigate the prevalence of cannabis among PWE seen at clinic, serum levels and gene expression of cytokines in these patients, and possibility that cannabis affects these cytokine levels
Tournebize et al. (77)	2019	Case report	1 PWE	To report a case of a child using illicit cannabis extract sold on the Internet for epilepsy treatment
Upadhyay et al. (78)	2017	Cross-sectional	Adults with epilepsy seen at American epilepsy center	To assess the prevalence of cannabis use in a tertiary care center's epilepsy clinic and identify factors potentially associated with its use
Von Wrede et al. (79)	2019	Cross-sectional	Adults with epilepsy attending German neurology ward or outpatient clinic	To capture the knowledge, expectations, and fears PWE have about medical cannabis
Wahby et al. (80)	2019	Cross-sectional	All patients receiving care in a Canadian epilepsy clinic	To determine whether the use of cannabis in PWE is associated with improved patient-reported indices of psychosocial wellbeing
Zafar et al. (81)	2021	Case series	10 PWE	To report on patients with severe, intractable, childhood-onset epilepsies using combined cannabinoid therapy
Zhu and Hazim (82)	2021	Cross-sectional	Parents of pediatric outpatients (age < 18 years old) with epilepsy seen at Hershey Medical Center	To characterize the prevalence, perceived effectiveness, and reasons for complementary and alternative medicine use among pediatric patients with epilepsy

AE, adverse effects; ASM, antiseizure medication; CUD, cannabis use disorder; EMU, epilepsy monitoring unit; NMC, non-medical cannabis; PWE, person/people with epilepsy; PNES, psychogenic non-epileptic seizure; QOL, quality of life; vEEG, video-electroencephalography.

**Table 2:** Publication identification and study characteristics between adult and pediatric studies.

	**All ages**	**Adult**	**Pediatric**
Number of total studies, *n* (%)	66 (100)	45 (68)	21 (32)
**Year**, ***n*** **(%)**			
2020–2022	15 (23)	9 (20)	6 (29)
2010–2019	43 (65)	28 (62)	15 (71)
2000–2009	4 (9.1)	4 (8.9)	0
1990–1999	2 (3.0)	2 (4.4)	0
<1990	2 (3.0)	2 (4.4)	0
**Country of origin**, ***n*** **(%)**			
USA	34 (52)	22 (49)	12 (57)
Canada	8 (12)	7 (16)	1 (4.8)
Germany	5 (7.6)	4 (8.9)	1 (4.8)
Australia	3 (4.6)	2 (4.4)	1 (4.8)
Egypt	2 (3.0)	2 (4.4)	0
UK	2 (3.0)	0	2 (9.5)
Argentina	1 (1.5)	1 (2.2)	0
Chile	1 (1.5)	0	1 (4.8)
France	1 (1.5)	0	1 (4.8)
Ireland	1 (1.5)	0	1 (4.8)
Italy	1 (1.5)	1 (2.2)	0
Jamaica	1 (1.5)	1 (2.2)	0
Lithuania	1 (1.5)	1 (2.2)	0
Mexico	1 (1.5)	0	1 (4.8)
Nigeria	1 (1.5)	1 (2.2)	0
Poland	1 (1.5)	1 (2.2)	0
South Africa	1 (1.5)	1 (2.2)	0
Spain	1 (1.5)	1 (2.2)	0
**Publication type**, ***n*** **(%)**			
Journal article	46 (70)	32 (71)	14 (67)
Abstract	18 (27)	12 (27)	6 (29)
Thesis	2 (3.0)	2 (4.4)	0
**Journal/conference**, ***n*** **(%)**			
Epilepsy and behavior	17 (26)	9 (20)	8 (38)
AAN annual meeting	6 (9.0)	5 (11)	1 (4.8)
AES annual meeting	2 (3.0)	2 (4.4)	0
Epilepsia	2 (3.0)	1 (2.2)	1 (4.8)
European congress on epileptology	3 (3.0)	2 (4.4)	1 (4.8)
JAMA	2 (3.0)	2 (4.4)	0
Other journals	25 (37.9)	19 (42.2)	6 (28.6)
Other conferences	7 (10.6)	3 (6.7)	4 (19.0)
University thesis	2 (3.0)	2 (4.4)	0
**Potentially significant funding/conflict of interest**, ***n*** **(%)**			
Yes	7 (11)	2 (4.4)	5 (24)
No	59 (89)	43 (96)	16 (76)
**Study type**, ***n*** **(%)**			
Cross-sectional	45 (68)	35 (78)	10 (48)
Case report/series	14 (21)	8 (18)	6 (29)
Qualitative	3 (4.6)	0	3 (14)
Cohort	3 (4.6)	1 (2.2)	2 (9.5)
Case-control	1 (1.5)	1 (2.2)	0
**Sample**, ***n*** **(%)**			
PWE	35 (53)	28 (62)	7 (33)
PWE using cannabis	23 (35)	9 (20)	14 (67)
Cannabis users	5 (7.6)	5 (11)	0
PWE and medical professionals	1 (1.5)	1 (2.2)	0
Medical professionals	1 (1.5)	1 (2.2)	0
Patients with neurological disorders	1 (1.5)	1 (2.2)	0
**Sample type**, ***n*** **(%)**			
Convenience	35 (53)	22 (49)	13 (62)
Consecutive	16 (24)	13 (29)	3 (14)
Snowball	2 (3.0)	1 (2.2)	1 (4.8)
Unknown	2 (3.0)	2 (4.4)	0
N/A (e.g., case report)	11 (17)	7 (16)	4 (19)
**Sample size, median (range)**			
Number of total study participants, median (range)	95 (1–657,072)	132 (1–657,072)	31 (1–378)
Number of PWE using NMC, median (range)	24.5 (1–37,945)	26 (1–37,945)	23.5 (1–272)

AACPDM, American Academy for Cerebral Palsy and Developmental Medicine; AAN, American Academy of Neurology; AES, American Epilepsy Society; CNSF, Canadian Neurological Sciences Federation; ILAE, International League Against Epilepsy; n, count; NMC, non-medical cannabis; PWE, people with epilepsy.

### 3.1. Publication identification and study characteristics

The following section is summarized in Table 1. Of the 66 included studies, 45 (68%) included only or mostly (i.e., >50%) adult participants (10, 11, 22, 24–28, 30, 32, 33, 36–39, 41–43, 46, 48–50, 52–63, 66–68, 71, 72, 75, 76, 78–80, 83), whereas 21 (32%) included only or mostly (i.e., >50%) pediatric participants (21, 23, 29, 31, 34, 35, 40, 44, 45, 47, 51, 64, 65, 69, 70, 73, 74, 77, 81, 82, 84). Most studies (88%), both adult and pediatric, were published after 2010 (10, 11, 21–29, 31, 32, 34, 35, 38–58, 60–66, 69–84). Most studies originated from the USA (52%) (10, 23, 27–31, 33, 39–42, 45, 46, 50–54, 58–65, 70–73, 78, 84), Canada (12%) (11, 21, 32, 36, 48, 49, 80), and Germany (7.6%) (21, 37, 38, 44, 68, 79). Most studies consisted in journal articles (68%) (10, 21, 23, 25–28, 30, 31, 33–40, 43–46, 48, 50–53, 57–59, 61, 62, 64–68, 70, 72–77, 79, 80, 84). The most popular journal was Epilepsy & Behavior, which published 17 (24%) studies in total (10, 11, 23, 25, 31, 39, 40, 44, 46, 53, 64, 65, 67, 72, 73, 75, 79, 84). Studies were most frequently cross-sectional (68%) (10, 11, 21–29, 31–33, 36–38, 40–44, 49, 50, 52–56, 61, 62, 64, 66–68, 71, 73, 75, 76, 78–80, 82–84) or case reports/series (21%) (30, 34, 39, 46–48, 51, 57, 58, 60, 63, 68, 77, 81). A few studies were qualitative (4.6%) (35, 70, 74) or longitudinal (6.1%) (45, 59, 65, 72). Most studies had samples consisting in PWE (71%) (10, 11, 21–23, 25, 30–34, 36, 38, 39, 41–44, 46, 49–51, 53–61, 63, 66, 67, 69, 70, 72, 74–83), though some specifically gathered samples of PWE who were known to use cannabis (17%) (29, 35, 40, 45, 47, 48, 64, 65, 71, 73, 84), and others gathered samples of cannabis consumers, searching for PWE among these consumers (7.6%) (26, 27, 37, 62, 68). Only non-random sampling methods were used; most frequently, convenience sampling (53%) (21, 24, 27, 29, 31–33, 35–37, 40, 41, 43–45, 48, 50, 52–55, 61, 62, 64, 67, 68, 71–75, 78, 81, 82, 84) was employed. The median sample size was 95 participants, but the range (1–657,072) was extremely wide due to the inclusion of case reports and two nationwide patient registry studies (50, 61). The median number of PWE using NMC was 24.5, once again with a very wide range (1–37,945) for the same reasons.

### 3.2. Demographics

Among the 20 studies providing explicit information on the age of adult PWE using NMC, all studies presented mean ages between 18 and 40 years or focused primarily on this age group (10, 11, 30, 32, 36, 38, 39, 41, 46, 53, 57, 58, 60, 61, 63, 71, 72, 74, 76, 80). As for pediatric studies, reported mean ages varied between 3 years and 11 years (21, 29, 31, 34, 45, 47, 51, 64, 69, 74, 77, 84). Sex was relatively balanced among pediatric studies, though in 14 out of 21 adult studies, there were slightly-to-moderately male-predominant samples (22, 30, 38, 39, 48, 50, 57, 58, 60, 61, 63, 71, 76, 80). In the seven studies detailing level of education, three had adult samples with mostly high school levels of education (36, 48, 80), three had adult samples with mostly post-secondary education (11, 41, 71), and one had a pediatric sample with most children attending mainstream schooling or special education classes (74). Ethnicity was explored in ten studies, with nine of these studies having predominantly Caucasian samples (25, 41, 45, 48, 50, 61, 70–72). Comorbidities were detailed in only eight studies, all of which were predominantly adult studies that explored various psychiatric comorbidities (11, 41, 48, 50, 61, 71, 72, 80). Socio-economic standing was rarely explored, though when it was, the method by which it was presented was variable, with some studies detailing employment rates and others mentioning median household income (11, 36, 41, 48, 50, 53, 71, 80). Marital status was only explored in three studies, two of which had participants who were predominantly single (48, 80), and one of which had participants who were predominantly married (71).

### 3.3. Data on epilepsy

Age at epilepsy onset was touched upon in 14 studies and was lower than 40 years in all but one case series (11, 30, 36, 39, 40, 45, 46, 51, 57, 60, 71, 74, 76, 77, 80, 84). Of the 13 studies reporting epilepsy type, four had samples with predominantly generalized-onset epilepsy (30, 32, 51, 60), and eight had samples with predominantly focal-onset epilepsy (36, 39, 48, 57, 63, 71, 76, 80). One other study consisted in a description of two cases, one of which had generalized-onset epilepsy and the other of which has focal-onset epilepsy (46). A total of 16 studies explored epilepsy syndromes; all nine pediatric studies had samples composed of children with epileptic encephalopathies though in variable proportions (21, 29, 40, 45, 51, 65, 69, 77, 84). The seven remaining adult studies detailed various epilepsy syndromes, such as idiopathic generalized epilepsy and temporal lobe epilepsy, without a clear pattern (11, 39, 48, 60, 63, 71, 75). One of these studies described psychogenic non-epileptic seizures in its sample (11). Use of ASMs was heterogeneously described in 19 studies, with some studies detailing the exact ASMs used, some indicating the proportion of PWE on polytherapy and monotherapy, and others specifying the number of failed ASM trials before NMC was begun (21, 30, 34, 36, 39, 40, 45, 46, 48, 51, 57, 58, 60, 72, 74, 76, 77, 80, 84). Given this heterogeneity, it was impossible to determine which ASMs were most frequently used. When the exact ASMs used were reported, these included phenytoin, phenobarbital, carbamazepine, zonisamide, levetiracetam, clobazam, topiramate, clonazepam, valproate, and vigabatrin (30, 39, 46, 51, 57, 58, 60, 77).

### 3.4. Data on NMC

#### 3.4.1. Experience

**Figure 3:**
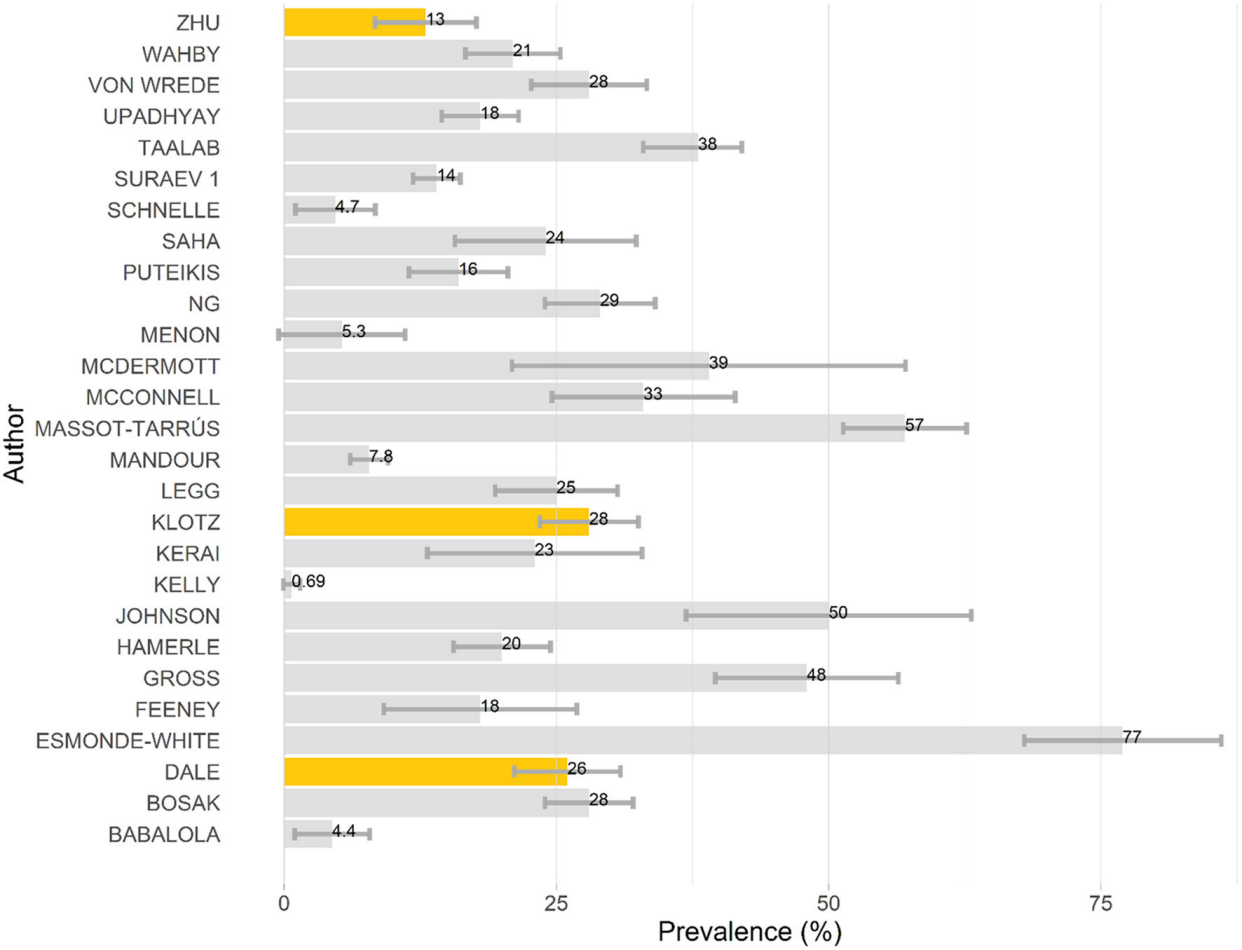
Lifetime prevalence of NMC use in PWE. This barchart presents the lifetime prevalence of NMC use in PWE for each publication for which this information was available. Gray bars represent prevalence calculated from adult samples, whereas orange bars represent prevalence calculated from pediatric samples. Confidence intervals are provided, as calculated using an α-error of 0.05.

The lifetime prevalence of NMC use in PWE ranged between 0.69 and 76.8% and can be visualized in Figure 3. When studies provided prevalence estimates without clearly specifying what type of prevalence they were (i.e., lifetime vs. active), their prevalence estimates were presumed to be lifetime prevalence. The active prevalence of NMC use in PWE ranged between 3.19 and 57.7% and can be visualized in Supplementary Figure S1. Two studies reported prevalence of “cannabis use disorder,” which were categorized as forms of active prevalence (50, 61). The prevalence of epilepsy in cannabis consumers was detailed in three adult studies reporting 7.2, 5.1, and 2.1%, respectively (26, 27, 37). Thirteen studies evaluated factors that were independently associated with cannabis use in PWE (10, 11, 27, 33, 36, 38, 50, 53, 61, 74, 75, 78, 80). The factors that were most frequently reported were as follows: younger adult (10, 11, 33, 38, 50, 53, 61, 78), male sex (10, 11, 38, 50, 61, 78, 80), and lower level of education (11, 78, 80). Dependence was only detailed in two studies, which characterized 1.4 and 3% of their respective sample of PWE as being dependent on cannabis (22, 36). Consequences of NMC consumption on mental, physical, and social spheres were variably detailed in seven studies (32, 41, 43, 45, 48, 72, 80). Recurrent consequences included stigma (32, 43) and higher levels of depression (41, 80), though one study also reported lower levels of depression (72).

#### 3.4.2. Habits

A total of 19 studies presented information on the type of NMC consumed by PWE (10, 21, 31, 39, 40, 45, 47, 51, 62, 63, 65, 69, 70, 72–74, 77, 81). The 14 pediatric studies all reported predominantly CBD-based NMC (21, 31, 40, 45, 47, 51, 65, 69, 70, 72–74, 77, 81), whereas the five adult studies reported more mixed findings (10, 39, 62, 63, 76), with one study even reporting that up to 82.5% of its participants used NMC of unknown composition (76). As for NMC administration methods, in all nine pediatric studies, NMC was primarily taken orally (31, 34, 45, 47, 51, 65, 69, 74, 77), whereas in nine of the 15 adult studies, NMC was predominantly smoked (10, 11, 30, 39, 41, 48, 71, 76, 80). The 16 studies exploring NMC dosage presented their data in vastly different methods (e.g., mg/kg/day, g/day, weekly, per consumption), rendering direct comparisons between studies unachievable (10, 11, 21, 32, 39, 40, 45, 48, 51, 57, 70, 72, 74, 76, 81, 84). Seven adult studies detailed the frequency of NMC use; in these studies, participants mostly used NMC daily or at least multiple times per week (10, 11, 30, 39, 41, 71, 76). No data on the frequency of NMC use could be directly extractable from the pediatric studies. The time since the start of NMC consumption and the age at which NMC consumption began were rarely stated and, when stated, yielded varying results (21, 30, 39, 40, 44, 48, 57, 63, 65, 76). Sources of NMC acquisition were listed in 17 studies and included, to varying degrees, the following: medical/recreational dispensaries, online shops, street vendors, friends and family, pharmacies, and homegrown NMC (10, 11, 29, 31, 32, 34, 35, 41, 44, 45, 48, 57, 69, 75–77). Only five studies detailed the price of NMC acquisition; each price was presented in a different currency in different time periods, rendering comparisons between studies difficult due to inflation (35, 70, 72, 74, 81).

#### 3.4.3. Beliefs

In the eight pediatric studies reporting on the goals of NMC consumption, seizure control was overwhelmingly the primary objective (34, 35, 44, 51, 64, 70, 74, 82). In contrast, in the 13 adult studies reporting on the goals of NMC consumption, seizure control and recreational use were both often cited as primary objectives (10, 11, 32, 36, 38, 39, 41, 57, 66, 71, 72, 75, 79). Across 11 studies, information sources for NMC were variable and included word-to-mouth, traditional/social media, and health professionals (24, 28, 32, 33, 35, 41, 44, 51, 57, 58, 70, 74). Variable proportions of participants discussed their NMC use with health professionals (24, 28, 32, 33, 41, 44, 51, 74). In half of studies exploring general knowledge of cannabis, participants had the general conception that NMC was more natural and/or safer than antiseizure medications (32, 35, 43, 66). Seven studies explored PWE's opinions on regulatory policies, but these opinions touched upon different aspects of NMC regulation and were too heterogeneous to be aptly summarized (10, 35, 52, 65, 70, 72, 74). Of the 33 studies detailing the perceived effects of NMC on seizures, 32 reported some beneficial effects on seizure control (10, 11, 21, 29–31, 34–36, 38–41, 45, 47, 48, 51, 54, 56–58, 60, 63, 65, 72–76, 78, 81, 84), whereas nine reported some detrimental effects on seizure control (11, 21, 29, 31, 38, 40, 65, 72, 74, 76). In only two of these nine studies did more participants perceive NMC to be detrimental than beneficial on seizure control (29, 38). As for other potential effects of NMC, ten studies reported varying rates of adverse effects, most commonly increased/decreased appetite, somnolence, various gastrointestinal symptoms, and irritability, (11, 21, 29, 31, 36, 40, 46, 65, 74, 84), whilst 14 studies mentioned various other benefits, such as improved mood, better sleep, lower levels of stress, and higher levels of alertness (11, 21, 31, 35, 36, 40, 47, 48, 65, 72, 74, 75, 81, 84).

## 4. Discussion

In the last years, there has been a steady rise in interest in the use of cannabis in epilepsy, with many studies reporting that CBD extracts may provide beneficial effects for seizure control (2, 3). In several countries, medical, pharmaceutical-grade cannabis may be prescribed by health professionals for seizure control in specific populations of PWE (2). Notwithstanding, personal experience and anecdotal accounts support the notion that a significant portion of PWE acquire cannabis by other means and that many of these PWE use cannabis in the hopes that it may diminish their seizure frequency and severity. This phenomenon is compounded by the fact that recreational cannabis itself is also being legalized in many parts of the world, and cannabis may therefore be more easily accessible in these areas regardless of its medical or recreational use (2). In addition, where cannabis is still bought from illicit vendors, one may question the quality and components of these cannabis products, as their production and distribution are not standardized (85). In this scoping review, we summarized what is known in the literature about the use of NMC (i.e., cannabis not obtained by prescription nor through special access programs) in PWE, focusing primarily on the lived experience, habits, and beliefs of these people regarding NMC. In the following sections, we will further analyze the literature and highlight knowledge gaps that may benefit from more research.

The evidence surrounding NMC use in PWE can be characterized as somewhat sparse and heterogeneous, with studies presenting varying samples, diverse aims, mostly low sample sizes, and differing outcome measures. The literature can naturally be divided into studies with adult samples and those with pediatric samples. Few relevant studies were published before the year 2000 (30, 33, 58, 59, 67, 68); no relevant pediatric studies were published before 2010, which may reflect how interest in cannabis use in children is more recent than in adults. Western countries were predominantly represented in the literature (10, 11, 21, 23, 25–34, 36–46, 48–54, 58–66, 68–75, 77–81, 84), and very little is known about NMC use in PWE from African, Middle Eastern, Central/Southern American, and Asian countries. Efforts should be made to obtain more data on the subject from non-Western countries. Regarding sample size, only two studies, which were nationwide patient registry studies (that unfortunately shared to some extent the same sample), had sample sizes in the 100,000s (50, 61). Otherwise, two studies had sample sizes of ~900 (75, 83), and every other study had samples sized at < 500 individuals. Oftentimes, PWE who used NMC only consisted in a subset of the overall sample and were therefore in even lower numbers. The majority of studies were cross-sectional by design (10, 11, 21–29, 31–33, 36–38, 40–44, 49, 50, 52–56, 61, 62, 64, 66–68, 71, 73, 75, 76, 78–80, 82–84), with only a few qualitative or longitudinal studies (35, 45, 65, 70, 74). Although cross-sectional studies appear to be a quick, cost-efficient way of evaluating PWE's experience with NMC, they may also be plagued by significant selection biases and response biases, especially when considering that studies may have been conducted in locations or in time periods where/when cannabis use was more stigmatized. Longitudinal or qualitative studies may provide different types of information whilst subverting some of the biases inherent to cannabis surveys, and more studies using these designs should be performed.

In terms of demographics, there seems to be a male preponderance for NMC use in adults with epilepsy (10, 11, 22, 30, 38, 39, 48, 50, 57, 58, 60, 61, 63, 71, 76, 78, 80). Such a finding, if confirmed, would probably reflect a general preponderance that men have for cannabis use vs. women (86). There are also slightly more cases of epilepsy in men than in women (87). Similarly, several adult studies suggested that a younger age may be associated with cannabis use in epilepsy (10, 11, 33, 38, 50, 53, 61, 78), though this may once more reflect an association that is independent of epilepsy itself (88). Interestingly, there is no literature focusing specifically on NMC use in elderly PWE. This constitutes a major knowledge gap, especially given the high prevalence of epilepsy in the elderly and the effects of cannabis on cognition (89, 90). In addition, little is known about NMC use in non-Caucasian PWE, as almost all studies that reported their sample's ethnicity reported a Caucasian predominance (25, 41, 45, 48, 50, 61, 70–72). The education level, comorbidities, socio-economic standing, and marital status of PWE using NMC were rarely explored, though some studies suggested associations between cannabis use in PWE and lower levels of education (11, 78, 80), psychiatric comorbidities (61, 80), a lower socio-economic standing (50, 53), and being single (80). To confirm these associations would require larger, more robust epidemiological studies.

Regarding the age at seizure onset, the type of epilepsy (focal- vs. generalized-onset), and the epilepsy syndromes at play, these data should always be disclosed in future research, mostly for better between-study comparability. We note no pattern from the literature regarding these factors, other than the fact that pediatric studies often presented samples of individuals with epileptic encephalopathies (21, 29, 40, 45, 51, 65, 69, 77, 84). No study specifically mentioned if children with tuberous sclerosis complex were included. How NMC use in individuals with epileptic encephalopathies evolves through time and particularly through adulthood remains poorly understood. Finally, data on ASM use was heterogeneously reported; how many failed ASMs were used before NMC was begun, which ASMs are most often taken with NMC, and how does the use of other ASMs evolve once NMC is begun all remain open questions. More data on ASMs would also allow for a better evaluation of the proportion of people with pharmacoresistant vs. pharmacosensitive epilepsy using NMC.

The prevalence of NMC use in PWE was extremely variable and depended upon the study populations at hand and methodological factors. Most studies reported lifetime prevalence of NMC use in PWE grossly between 10 and 40% (25, 31, 33, 38, 43, 44, 49, 53, 54, 59, 66, 67, 75, 76, 78–80, 82). The two largest studies (with sample sizes several 100,000s larger than every other study) reported prevalence of NMC use in PWE of < 10%, though these were prevalence for “cannabis use disorder” in people hospitalized with epilepsy (50, 61). As a matter of fact, in many studies, the type of prevalence being reported (e.g., lifetime cannabis use, active cannabis use, cannabis use only after epilepsy diagnosis) was ambiguous. Future studies exploring the prevalence of NMC use in PWE should carefully distinguish the type of prevalence they are studying. Very few studies reported the prevalence of epilepsy in cannabis consumers, and this may represent an avenue for future research (26, 27, 37). The consequences of NMC use on mental, physical, and social spheres were rarely explored, though recurrent themes included feelings of being stigmatized (32, 43) and depressed mood (41, 80). Given the prevalence of mood disorders in PWE, how cannabis consumption interacts with this association would be important to clarify (91, 92). Interestingly, though some studies used self-reported outcome scales to measure mood and quality of life (41, 45, 71, 72, 80), no study has used formal neuropsychological tests to evaluate the impact of NMC on PWE. Measures of dependence were almost never addressed in the literature and would be important to evaluate in future studies.

The main type of NMC used varied between pediatric and adult studies; children appeared to mostly use oral CBD extracts (21, 31, 40, 45, 47, 51, 65, 69, 70, 72–74, 77, 81), whereas adults used NMC with various CBD-THC compositions (10, 39, 62, 63, 76). The route of administration in adults was also much more varied, although smoking seemed to be a recurrent, predominant route (10, 11, 30, 39, 41, 48, 71, 76, 80). This difference in NMC use between age groups can probably be explained by the fact that children used NMC primarily for seizure control (34, 35, 44, 51, 64, 70, 74, 82), whereas adults often used NMC for recreational purposes as well (10, 11, 32, 36, 38, 39, 41, 57, 66, 71, 75, 79). No clear correlation between NMC type, NMC administration route, and goals of consumption has been established yet, and this potential association could benefit from confirmatory studies. The sources of NMC acquisition included both legal and illicit routes and were highly variable (10, 11, 29, 32, 34, 35, 41, 44, 45, 48, 57, 69, 75–77). More research on the importance and implications of illicit cannabis vendors in PWE should be done, especially given the potential variability in the quality of the cannabis sold by these vendors (85). Information on NMC dosage, frequency of consumption, and pricing was sparse and heterogeneous. The age at which PWE began consuming NMC was rarely explored, representing another significant knowledge gap. In the case of PWE consuming NMC for seizure control, it would be interesting to investigate if they had a history of recreational cannabis use before beginning NMC for seizure control. Ultimately, more research will simply need to be conducted which systematically evaluates the habits of PWE regarding their NMC use. Care will need to be taken to present data in a manner that can be readily compared between studies.

PWE receive information on NMC from various sources, many non-medical. These sources included the internet, social media (e.g., Facebook), traditional media (e.g., TV broadcasts), and friends and family (24, 28, 32, 33, 35, 41, 44, 51, 57, 58, 70, 74). On a public health standpoint, further investigation of PWE's sources of information—especially social media where information may be largely unregulated (93)—may help design awareness campaigns and targeted knowledge sharing strategies. Many PWE did not disclose their NMC use to their physicians, yet the reasons why this occurred are not well-understood (24, 28, 32, 33, 41, 44, 51, 74). Feelings of stigma or of being unsupported by physicians have been cited as barriers to disclosure (32, 35). PWE's general knowledge of cannabis and opinions on regulatory policies were not often explored, though there did seem to be a conception that cannabis was more natural than ASMs (32, 35, 43, 66). The majority of PWE across all studies perceived that NMC aided in seizure control as well as in other domains, such as cognition, sleep, mood, and anxiety (10, 11, 21, 29–31, 34–36, 38–41, 45, 47, 48, 51, 54, 56–58, 60, 63, 65, 72–76, 78, 81, 84). On numerous occasions, being able to wean off other ASMs was cited as an extra benefit of NMC use (11, 21, 29, 31, 36, 40, 46, 65, 74, 84), which begs the question of what proportion of PWE perceive NMC as an add-on therapy rather than a replacement therapy and vice versa. The perceived benefits of NMC use were probably distorted by confirmation biases, and further research should be carried out to distinguish between the objective and subjective effects of NMC as well as to explore what factors could predict a greater bias.

This scoping review featured some limitations. Firstly, given the sheer scope of this study, decisions had to be made to not explore certain variables that may have been interesting to explore. For example, the concomitant/past use of ketogenic diet, vagus nerve stimulation, and epilepsy surgeries could have been searched for in this review. Likewise, though this scoping review focused exclusively on PWE who used NMC, other populations could have been studied. For example, in the screening phase of this review, many studies focusing on healthcare professionals' experience with PWE using NMC were excluded. A future review tackling this subset of studies could yield interesting findings. Secondly, certain variables, such as opinions on regulatory policies, were in hindsight a bit vague and yielded wide-ranging results. Separating these variables into sub-topics would perhaps have enhanced the between-study comparability at the expense of feasibility. Finally, some may argue that the inclusion of case reports/series as primary sources of evidence renders our final interpretations less robust; however, the inclusion of these studies also allowed for a more comprehensive portrayal of the literature.

## 5. Conclusion

In this scoping review, we summarized the body of literature surrounding NMC use in PWE, with emphasis on PWE's experience, habits, and beliefs concerning NMC. Overall, the literature can be summarized as heterogeneous and mostly populated by small, cross-sectional studies for which selection, participation, and confirmation biases are of concern. Though some topics are better explored than others, significant knowledge gaps exist throughout the literature and include (but are not limited to) the use of NMC in PWE from non-Western countries, the patterns of use of other ASMs in concomitance with NMC, NMC consumption in elderly PWE, the NMC consumption patterns of PWE (including the influence of illicit vendors), the barriers to the disclosure of NMC use to physicians, the comorbidities and characteristics of consumers vs. non-consumers, and the neuropsychological effects of NMC consumption in PWE. We hope our work may encourage researchers to address these gaps through high-quality research. We equally hope to encourage researchers to explore the landscape of NMC use in other neurological conditions, such as multiple sclerosis, Alzheimer's disease, and amyotrophic lateral sclerosis.

## Data availability statement

The original contributions presented in the study are included in the article/Supplementary material, further inquiries can be directed to the corresponding author.

## Author contributions

JL: study design, data collection, data analysis, and drafting. CA: data collection and data analysis. DT: data collection and manuscript revision. DC and CD: manuscript revision. DJ-A and MK: study design and manuscript revision. DN: study design, manuscript revision, and principal investigator. All authors contributed to the article and approved the submitted version.
