# Supplementary material for: Use of non-medical cannabis in epilepsy: A scoping review
Source: Front Neurol. 2023 Mar 6;14:1132106. doi: 10.3389/fneur.2023.1132106 (PMC10025318; doi:10.3389/fneur.2023.1132106)
Supplement: Supplementary file 1 [file Table_1.PDF]

Supplementary Material

Use of Non-Medical Cannabis in Epilepsy: A Scoping Review

Jimmy Li [*], Cassandra C. Areal, Dènahin Hinnoutondji Toffa, Daphné Citherlet, Charles Deacon, Didier Jutras-Aswad, Mark Robert Keezer, and Dang Khoa Nguyen

*** Correspondence:** Jimmy Li: jimmy.li@umontreal.ca

Database	Search query
OVID Medline<br>1947 – 2022	((cannabis or marijuana or hemp or hashish or marihuana or bhang or ganja).ab,ti,kw. or cannabis.sh.) and ((epilep* or seizure* or convuls* or infantile spasm*).ab,ti,kw. or epilepsy.sh.) not (animals not humans).sh.
OVID Embase<br>1974 – 2022	#1 ((cannabis or marijuana or hemp or hashish or marihuana or bhang or ganja).ab,ti,kw. or cannabis.sh.) and ((epilep* or seizure* or convuls* or infantile spasm*).ab,ti,kw. or epilepsy.sh.) not (animal not human).sh.<br>#2 Limit #1 to medline<br>#3 #1 not #2
OVID APA PsycInfo<br>1806 – 2022	#1 ((cannabis or marijuana or hemp or hashish or marihuana or bhang or ganja).mp. or cannabis.hw,mh.) not (animal not human).po.<br>#2 ((epilep* or seizure* or convuls* or infantile spasm*).mp. or epilepsy.hw,mh.) not (animal not human).po.<br>#3 #1 and #2
Web of Science<br>1975 – 2022	TS=(cannabis or marijuana or hemp or hashish or marihuana) AND TS=(epilep* or seizure* or convuls* or infantile spasm*)
Networked Digitalized Library of Theses and Dissertations (NDLTD)	(cannabis OR marijuana OR hemp OR hashish OR marihuana) AND (epilepsy OR seizure)
Metalib U.S. Government Publishing Office	#1 cannabis AND epilepsy<br>#2 marijuana AND epilepsy
New York Academy’s Grey Literature Report	(cannabis OR marijuana OR hemp) AND epilepsy

ClinicalTrials.gov	cannabis AND epilepsy
Canada’s Drug and Health Technology Agency	cannabis AND epilepsy
Epilepsy Society	cannabis
Addiction Group	epilepsy
American Epilepsy Society	cannabis OR marijuana OR hemp OR hashish OR marihuana
NLM catalog	(cannabis or marijuana or hemp or hashish or marihuana) and (epilepsy or seizure disorder)

Table S1. Detailed search strategy

	All ages, n (%)	Adult, n (%)	Pediatric, n (%)
# total studies	66 (100)	45 (68)	21 (32)
**Demographics data**			
# studies reporting age	33 (50)	21 (47)	12 (57)
# studies reporting sex	32 (48)	21 (47)	11 (52)
# studies reporting level of education	7 (11)	6 (13)	1 (4.8)
# studies reporting ethnicity	10 (15)	8 (18)	2 (9.5)
# studies reporting comorbidities	8 (12)	8 (18)	0
# studies reporting socio-economic level	8 (12)	8 (18)	0
# studies reporting marital status	3 (4.5)	3 (6.7)	0
**Epilepsy data**			
# studies reporting age at onset	14 (21)	8 (18)	6 (29)
# studies reporting epilepsy type	13 (20)	12 (27)	1 (4.8)
# studies reporting epilepsy syndrome	16 (24)	7 (16)	9 (43)
# studies reporting ASM use	19 (29)	11 (24)	8 (38)
**NMC data – Experience with NMC**			
# studies reporting lifetime prevalence of NMC use in PWE	28 (42)	24 (53)	4 (19)
# studies reporting active prevalence of NMC use in PWE	10 (15)	9 (20)	1 (4.8)
# studies reporting prevalence of epilepsy in NMC users	3 (4.5)	3 (6.7)	0
# studies reporting factors associated with NMC use	13 (20)	12 (27)	1 (4.8)
# studies reporting dependency	2 (3.0)	2 (4.4)	0

# studies reporting consequences of NMC use on physical, mental, and social spheres	7 (11)	6 (13)	1 (4.8)
**NMC data – Habits with NMC**			
# studies reporting NMC type	19 (29)	6 (13)	13 (62)
# studies reporting administration method	24 (36)	15 (33)	9 (43)
# studies reporting dose	16 (24)	8 (18)	8 (38)
# studies reporting frequency of use	7 (11)	7 (16)	0
# studies reporting time since start of use	10 (15)	6 (13)	4 (19)
# studies reporting age at first use	4 (6.1)	3 (6.7)	1 (4.8)
# studies reporting source of acquisition	17 (26)	8 (18)	9 (43)
# studies reporting product pricing	5 (7.6)	1 (2.2)	4 (19)
**NMC data – Beliefs regarding NMC**			
# studies reporting goals for consumption	21 (32)	13 (29)	8 (38)
# studies reporting information sources	11 (17)	6 (13)	5 (24)
# studies reporting general knowledge of cannabis	8 (12)	5 (11)	3 (14)
# studies reporting opinion on regulatory policies	7 (11)	3 (6.7)	4 (19)
# studies reporting perceived effects on seizures	33 (50)	18 (40)	15 (71)
# studies reporting perceived other effects	20 (30)	9 (20)	11 (52)

Table S2: Demographics, NMC data, and epilepsy data between adult and pediatric studies

n = count; NMC = non-medical cannabis; PWE = people with epilepsy.

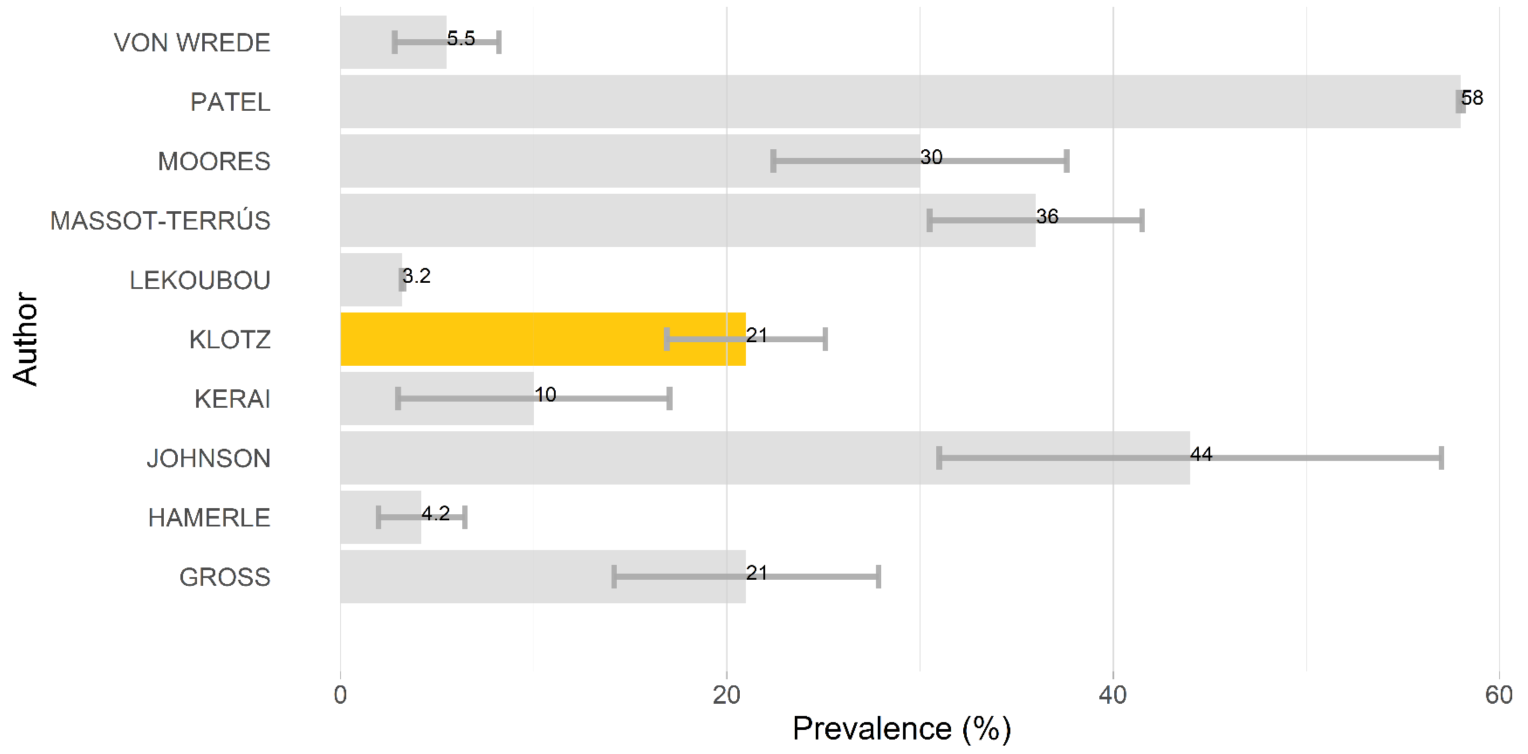

Figure S1: Active prevalence of NMC usage in PWE

This barchart presents the active prevalence of NMC usage in PWE for each publication for which this information was available. Grey bars represent prevalence calculated from adult samples, whereas orange bars represent prevalence calculated from pediatric samples. Confidence intervals are provided, as calculated using an α-error of 0.05.

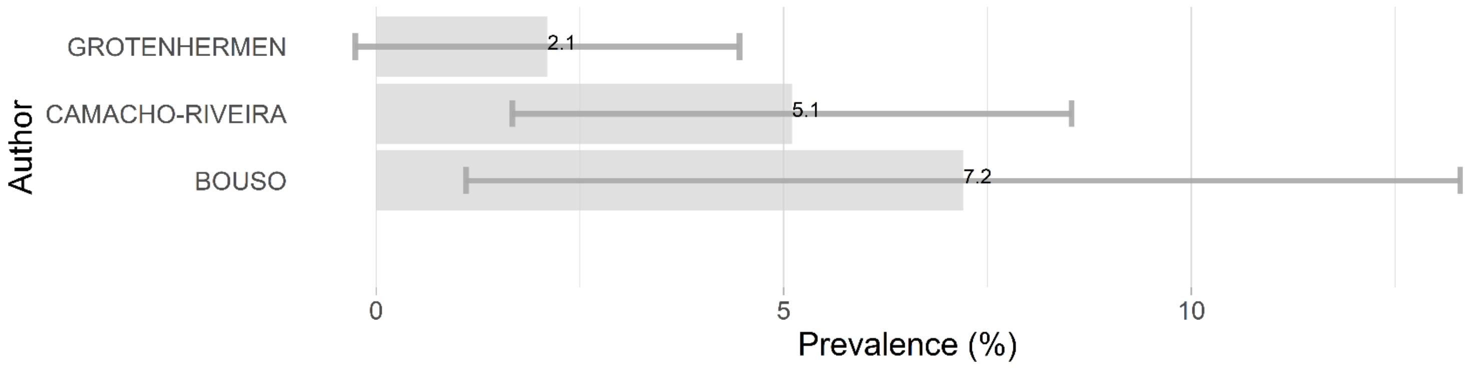

Figure S2: Lifetime prevalence of epilepsy in cannabis consumers

This barchart presents the lifetime prevalence of epilepsy in people consuming cannabis for each publication for which this information was available. All three publications were based on adult samples. Confidence intervals are provided, as calculated using an α-error of 0.05.
